# Noncovalent Conjugates of Anthocyanins to Wheat Gluten: Unraveling Their Microstructure and Physicochemical Properties

**DOI:** 10.3390/foods13020220

**Published:** 2024-01-10

**Authors:** Ziqi Guo, Jian Huang, Xin Mei, Yong Sui, Shuyi Li, Zhenzhou Zhu

**Affiliations:** 1 School of Modern Industry for Selenium Science and Engineering, Wuhan Polytechnic University, Wuhan 430023, China; guoziqi0501@126.com (Z.G.); jianhuang202202@163.com (J.H.); 2 College of Food Science and Engineering, Wuhan Polytechnic University, Wuhan 430023, China; 3 Key Laboratory for Deep Processing of Major Grain and Oil, Ministry of Education, Wuhan Polytechnic University, Wuhan 430023, China; 4 National R&D Center for Se-Rich Agricultural Products Processing Technology, Wuhan Polytechnic University, Wuhan 430023, China; 5 Institute for Farm Products Processing and Nuclear-Agricultural Technology, Hubei Academy of Agricultural Science, Wuhan 430064, China; liangshijiagong@126.com (X.M.); suiyong_1231@hbaas.com (Y.S.)

**Keywords:** grape skin anthocyanin extract, gluten protein, microstructure and physicochemical properties

## Abstract

Intake of polyphenol-modified wheat products has the potential to reduce the incidence of chronic diseases. In order to determine the modification effect of polyphenols on wheat gluten protein, the effects of grape skin anthocyanin extract (GSAE, additional amounts of 0.1%, 0.2%, 0.3%, 0.4%, and 0.5%, respectively) on the microstructure and physicochemical properties of gluten protein were investigated. The introduction of GSAE improves the maintenance of the gluten network and increases viscoelasticity, as evidenced by rheological and creep recovery tests. The tensile properties of gluten protein were at their peak when the GSAE level was 0.3%. The addition of 0.5% GSAE may raise the denaturation temperature of gluten protein by 6.48 °C–9.02 °C at different heating temperatures, considerably improving its thermal stability. Furthermore, GSAE enhanced the intermolecular hydrogen bond of gluten protein and promoted the conversion of free sulfhydryl groups to disulfide bonds. Meanwhile, the GSAE treatment may also lead to protein aggregation, and the average pore size of gluten samples decreased significantly and the structure became denser, indicating that GSAE improved the stability of the gluten spatial network. The positive effects of GSAE on gluten protein properties suggest the potential of GSAE as a quality enhancer for wheat products.

## 1. Introduction

People’s diet structure and pace of life have changed dramatically in the last decade as living conditions have improved substantially. Consequently, the prevalence of metabolic chronic illnesses such as obesity, diabetes, cardiovascular disease, and cancer were increased, which pose a severe danger to the nation’s health. Consumers have raised their expectations for food’s nutritional worth as their awareness of a healthy diet has grown. Including biologically active substances in food, such as phenolic compounds, is a successful strategy to minimize the prevalence of chronic illnesses [1,2]. Therefore, many phenolic compounds, such as grape seed extracts and tea catechins, were enriched in wheat flour-based products [3,4]. Encouragingly, numerous research has proved that the interaction between phenolic chemicals in wheat flour and gluten protein is beneficial for the modification of both. On the one hand, phenolic substances may elevate the antioxidant activity of gluten protein, which helps to regulate blood sugar levels in the body [5,6,7]; on the other hand, interactions with proteins have the potential to enhance phenols’ thermal stability, decrease their degradation rate, and improve their bioavailability in vivo [8,9].

In the past, several studies have emphasized the importance of value-added wheat products with the supplementation of natural food components to increase the nutritional value as well as make it more acceptable for the health-conscious population [10,11,12]. In recent years, a variety of nutritionally fortified wheat products have been produced, using modern processing techniques to combine different nutritional and health ingredients with wheat flour. These include green seaweed noodles [13], *Tinospora cordifolia* cookies [11], which are fortified with plant and animal ingredients, dietary fiber noodles [14], tea polyphenols bread [15], and soy protein hydrolysates noodles [16], which are fortified with functional factors. For example, Xu, Guo, Roman, Pico and Martinez [12] found that bread prepared with a certain amount of okra seed and seedless pods increased the dietary fiber and phenolic content of the product considerably, reducing starch digestibility and controlling blood sugar.

Grapes contain numerous phenolics consisting of flavonoids (including anthocyanins) and non-flavonoids (stilbenes, hydroxybenzoic, and hydroxycinnamic acids) [17,18]. Grape skin, a byproduct of grapes, is mostly composed of anthocyanins. There is a growing interest in anthocyanins because they may be good for health as dietary antioxidants and to help prevent cancer, diabetes, inflammation, and neurological or cardiovascular diseases [19,20]. The antioxidant capacity of anthocyanins makes them suitable candidates for use in conferral of resistance to atherosclerosis [20]. Therefore, adding anthocyanins to food may be beneficial to human health. Products made from wheat flour may benefit from the addition of grape skin anthocyanin extract (GSAE), which could enhance their functional properties and aid in the avoidance of chronic diseases. However, in our preliminary study [8], we found that the gliadin inside wheat gluten (WG) interacts with GSAE, but its potential mechanism with WG was unclear.

Polyphenol–protein interaction is an important research direction in the area of protein modification and food processing. Some previous studies verified that polyphenol showed the potential of interplaying with gliadin via both hydrophobic interactions and the modification of secondary structures [21,22]. Therefore, the aim of the present research was to investigate the effects of GSAE on the network structure, rheological properties, thermal stability, and microstructure of gluten, and to explore the possible mechanism. The results of this study will provide further guidance for the application of polyphenol compounds in flour products, and propose possible research directions to lower the prevalence of chronic diseases.

## 2. Materials and Methods

### 2.1. Materials

Wheat gluten (purity 85.7%) was purchased from Henan Lianhua Monosodium Glutamate Group Co., Ltd. (Zhoukou, China). Grape skin extracts were purchased from Yunnan Tonghai Yang Natural Products Co., Ltd. (Yuxi, China). The macroporous resin AB-8 was obtained at Shanghai Macklin Biochemical Co., Ltd. (Shanghai, China). 5′-Dithiobis-(2-nitrobenzoic acid) (DTNB) and anilino-1-naphthalenesulfonic acid (ANS) were purchased from Aladdin Reagent Co., Ltd. (Shanghai, China). Tris, Glycine, SDS, Acetic Acid, KBr, NaCl, and Ethylene Diamine Tetraacetic Acid (EDTA) were purchased from Sinopharm Chemical Reagent Co., Ltd. (Shanghai, China). All the other reagents of analytical grade were purchased from Sinopharm Chemical Reagent Co., Ltd. (Beijing, China). Experimental water was obtained using a Milli-Q^®^ Advantage A10 water purification system (Merck KGaA, Darmstadt, Germany).

### 2.2. Extraction and Purification of Anthocyanin

The extraction and purification of GSAE were performed as described in the previous procedure [8]. Briefly, GSAE solution (200 mL, pH 3) was added with a flow rate of between 3 and 4 BV/h to a 400 mL pretreatment AB-8 macroporous resin column. Then, using 70% (*v*/*v*) ethanol with a pH of 3 and a flow rate of between 1 and 2 BV/h, a solution rich in anthocyanins was produced. After removed the organic solvent by rotary evaporation at a temperature of 45 °C, the concentrated solution was collected and freeze-dried (ALPHA2-4 LD plus, Christ, Germany). The components of GSAE were analyzed by HPLC-MS^2^. The results showed that the main components of GSAE were delphinidin-3-O-glucoside (D3G), cyanidin-3-O-glucoside (C3G), petunidin-3-glucoside (Pt3G), peonidin-3-glucoside (Pn3G), and malvidin-3-O-glucoside (M3G) [8].

### 2.3. Preparation of WG-GSAE Sample

The WG-GSAE sample was prepared using a dough mixer (HM740, Hauswirt, Qingdao, China). The GSAE solution (1 mg/mL) was added to the gluten protein sample so that the final amount of GSAE was 0, 0.1, 0.2, 0.3, 0.4, and 0.5%. The dough was prepared according to the method of Chen, et al. [23]. Briefly, the flour mix (100 g) was added with 40 mL of distilled water and mixed until the dough was formed. The dough was then heated in a water bath at 30 °C, 50 °C, 70 °C, and 90 °C for 30 min.

### 2.4. Rheological Measurement

According to the method of Shah, et al. [24], the rheological properties of gluten dough were determined by a rheometer (Discovery HR-1, TA Instruments, New Castle, DE, USA) using parallel plates spaced 2 mm apart (diameter 20 mm). The dough was stabilized for 10 min before placing the plate sensor and used a scraper to scrape off excess dough. The edges of the dough were sealed with silicone oil to prevent moisture loss. A frequency scan of 0.1 to 100 Hz was carried out at a strain of 0.02% (in a linear viscoelastic zone) at a temperature of 25 °C. The changes in the energy storage modulus (G′) and loss modulus (G″) of gluten protein under frequency scanning were analyzed.

### 2.5. Thermal Properties Analysis

Differential scanning calorimetry (DSC) measurements were performed with a DSC TA-Q2000 (TA Instruments, New Castle, DE, USA) according to the method of Liu, et al. [25]. Briefly, the sample (2–3 mg) was sealed in an aluminum dish, loaded into the equipment room, and then heated from 30 °C to 150 °C at a heating rate of 10 °C/min. The enthalpy change (ΔH) was obtained from the DSC thermogram.

### 2.6. Tensile Properties Measurement

The tensile properties of gluten dough were measured using an extensograph according to GB/T 14615-2019 [26]. The extensibility (mm), curve area (cm^2^), maximum elongation resistance (EU), elongation resistance (EU_50_) at 50 mm, and elongation ratio of the dough were recorded.

### 2.7. Creep and Recovery Properties Measurement

The gluten protein creep properties were measured using a dynamic rheometer according to the method of Bockstaele et al. [27]. The operating parameters were as follows: a 40 mm fixture plate with a gap of 2 mm, a shear stress of 100 Pa at 25 °C, and a creep and recovery time of 5 min. The experiment starts with creep and then proceeds with recovery. The experimental data were fitted with the Burgers model:

$$J=f(t)=J_{0}+J_{1}\left(1-EXP\left(\frac{-t}{\lambda_{ret}}\right)\right)+\frac{t}{\mu_{0}}$$

where J_0_ is the instantaneous creep compliance; J_1_ is the hysteretic creep compliance; t is the sample relaxation time; λ_ret_ is the mean relaxation time; and μ_0_ is the viscosity factor.

### 2.8. Free Sulfhydryl (SH) and Disulfide Bond (S-S) Content

The content of free SH was determined by the method of Luo et al. [28] with some modifications. The gluten sample (50 mg) was suspended in 10 mL 0.2 M Tris-HCl buffer (pH 8.0, 8 M urea, 1% SDS, and 3 mM EDTA) and reacted for 30 min, followed by centrifuging at 9600× *g* for 20 min and the supernatant was collected. Subsequently, the mixture (4 mL) was reacting with 0.1 mL 0.04 mg/mL Ellman reagent (5,5′-dithiobi-2,2′-nitrobenzoic acid) at room temperature for 30 min. The absorbance of the diluted supernatant was read at 412 nm with the reagent buffer as blank. The content of free sulfhydryl groups in the sample is calculated as follows:

$$Free\;SH\;content\;(\mu M\;SH/g)=\frac{73.53\times A_{412}\times D}{C}$$

where “73.53” is a constant, A_412_ is the absorbance of the sample at 412 nm, D is dilution factor, and C is the protein concentration (mg/mL).

The supernatant (1 mL) after centrifugation mentioned above was added into 4 mL 0.2 M Tris-HCl buffer and 0.1 mL β-mercaptoethanol. After 1 h of warming in a 25 °C water bath, 10 mL of 12% trichloroacetic acid solution was added. The mixture was kept in a water bath at 25 °C for 1 h, centrifuged at 8000 r/min for 20 min, and the precipitate was re-suspended with trichloroacetic acid for 3 times. Tris-HCl buffer (10 mL 0.2 M) was added to redissolve samples, and then 0.1 mL Ellman reagent was added. After the color reaction was carried out at room temperature for 20 min, the absorbance was determined at a 412 nm wavelength with distilled water as a blank. The total SH content of the sample was calculated according to the method of free SH. The content of the gluten S-S bond is calculated as follows:

$$The\;\mathrm{S-S}\;bond\;content\;(\mu mol/g)=\frac{The\;total\;SH-The\;free\;SH}{2}$$

### 2.9. Surface Hydrophobicity

The surface hydrophobicity of gluten samples was measured according to the method of Li, et al. [29] with some modifications. Briefly, gluten (400 g) was introduced to 20 mL 0.01 M phosphate buffer (pH 7.0) and stirred for 2 h, followed by centrifuging 8000× *g* for 10 min. The 4 mL diluted sample was mixed with 50 μL 8-aniline-1-naphthalene sulfonic acid (ANS) and its fluorescence intensity was measured in a fluorescence spectrophotometer (F-4600, Hitachi, Japan). The excitation and emission wavelengths are 370 nm and 490 nm, respectively, and the slit width is 5 nm. The surface hydrophobicity of gluten corresponds to the slope of fluorescence intensity to protein concentration.

### 2.10. Scanning Electron Microscope (SEM) Analysis

After freeze-drying using an ALPHA2-4 LD plus freeze-dryer (Christ, Germany), the gluten protein samples were adhered to the conductive tape, and the microstructure was examined using a SEM (S-3000N, Hitachi, Japan) after gold was sprayed on the surface.

### 2.11. Statistical Analysis

Each parameter was measured in triplicate and all the data were presented as mean ± standard deviation. Data were subjected to analysis of variance (ANOVA). The statistical significance was identified at the 95% confidence level (*p* < 0.05) and was calculated by SPSS 26.0 (Chicago, IL, USA) software. Origin 2018 (Origin-Lab, Northampton, MA, USA) software was used for data processing and chart plotting.

## 3. Results and Discussion

### 3.1. Rheological Properties

**Figure 1:**
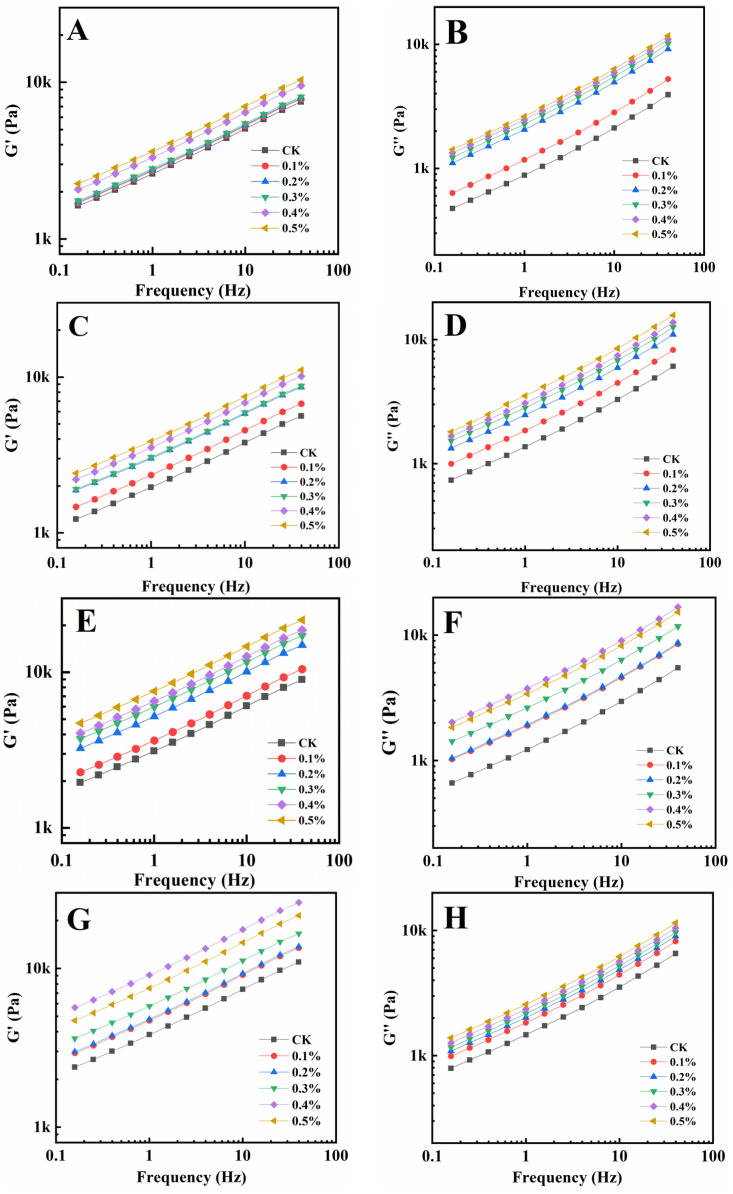
The relationship between G′ and G″ and frequency of gluten protein samples heated at 30 °C (**A**,**B**), 50 °C (**C**,**D**), 70 °C (**E**,**F**), and 90 °C (**G**,**H**) with different GSAE dosages, respectively.

Rheological qualities provide information on the viscoelasticity of gluten and have connections to processing parameters and food quality [30,31]. The energy storage modulus (G′) represents the amount of energy recovered per deformation cycle, which reflects the elasticity of gluten solids, while the loss modulus (G″) represents the amount of energy dissipated as heat per deformation cycle, which reflects the viscosity response of the dough [32,33]. Figure 1 shows the relationship between G′ and G″ and the frequency of gluten protein samples heated at 30 °C, 50 °C, 70 °C, and 90 °C with different GSAE dosages, respectively. It could be observed that following GSAE treatment, both G′ and G″ of gluten increased with increasing frequency. On the whole, G′ and G″ increased with the increase of GSAE content. Gluten plays a major role in the rheological properties of flour dough, and the increase in viscoelasticity of gluten indicates that the addition of GSAE enhances the strength and gluten network of the dough [34]. And as a typical viscoelastic material, the G′ of gluten is greater than its G″ in all circumstances, showing solid-like behavior as previously reported [35]. According to research, heating gluten protein from 30 °C to 50 °C resulted in a reduction in G′, whereas heating it over 50 °C resulted in a strengthening of protein structure and an increase in G′. It was consistent with the results of Mehmet et al., Hayta and Schofield [36], which suggested that non-covalent cross-linking of macromolecules of glutenin might occur when the temperature is raised 50 °C. Furthermore, the molecular weight of the gluten protein polymer and its distribution, as well as the features of high molecular weight glutenin subunits, are important variables influencing the elastic modulus of gluten protein. The G″, on the whole, slightly declined as the temperature rose from 30 °C to 90 °C, which might be attributed to the decrease in extensibility of wheat prolamin following heat induction. When the treatment temperature was too high, many groups in the protein molecules were activated, and immediately interacted with the surrounding proteins to form protein aggregation particles, which were not easy to form a continuous three-dimensional network structure, and ultimately led to high hardness and poor extensibility of the dough [37]. The G′ and G″ increase as the quantity of GSAE added increases, showing that GSAE interacts with gluten proteins and enhances viscoelasticity, particularly at high temperatures, which may facilitate aggregation, indicating the possible prospect of GSAE processing applications.

### 3.2. Thermal Properties

**Figure 2:**
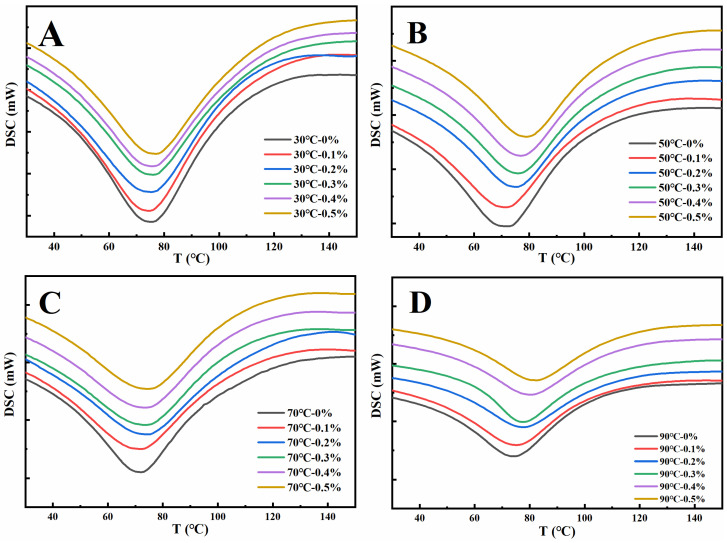
The differential scanning calorimetry curve of gluten protein treated with different levels of GSAE at the heat temperature of 30 °C (**A**), 50 °C (**B**), 70 °C (**C**), and 90 °C (**D**).

The thermal denaturation of proteins refers to the process in which the structure of peptide chains undergoes rearrangement during heating, resulting in a naturally ordered state changing to a disordered state. Denaturation temperature (Tp) and denaturation enthalpy (ΔH) are the main parameters to characterize the thermal denaturation process of proteins. The Tp reflects the thermal stability of the protein, while the ΔH change reflects the hydrophilic/hydrophobic nature of the protein molecule, and also reflects the degree of aggregation of the protein molecule [38]. As depicted in Figure 2, during the heating process, the denaturation peaks of the gluten samples were all downward, demonstrating that the gluten samples of different treatments absorbed heat during the heating denaturation process [39]. The Tp and ΔH of gluten protein in Table 1 can be obtained from Figure 2. As shown in Table 1, increasing the amount of GSAE from 0% to 0.5% raised the denaturation temperature of gluten protein at 30 °C, 50 °C, 70 °C, and 90 °C by 4.0 °C, 7.1 °C, 6.5 °C, and 7.1 °C, respectively, indicating that the thermal stability of gluten protein was improved after the introduction of GSAE. The interactions between gluten and GSAE were most likely the result of a hydrophobic interaction, which reduced heat-induced denaturation [40]. Furthermore, as the heating temperature increased from 30 °C to 90 °C, ΔH in Control (CK) reduced from 216.6 J/g to 160.5 J/g (Table 1), indicating that the degree of protein aggregation increased during the whole heating process [36]. The reduction of ΔH reflects a shift in protein structure from ordered to disordered [38]. Under different heating temperatures, the ΔH of gluten protein decreased with the increase in the GSAE concentration, which might contribute to the process of heat denaturation destroying the hydrophobic interaction between GSAE and proteins and thus releasing heat [39].

### 3.3. Tensile Properties

The tensile property of dough refers to the elasticity and plasticity of dough after deformation and disappearance of the external force [41]. The tensile properties of gluten are shown in Table 2. When the GSAE concentration was 0.3%, the stretching energy, breaking distance from 30 min, fracture energy, and extensibility of gluten protein reached the highest as 24.1 ± 1.0 g, 65.3 ± 1.9 mm, 808.0 ± 5.0 g, and 61.6 ± 5.1 mm, respectively, which were significantly (*p* < 0.05) higher than CK, indicating that the gluten protein sample under 0.3% GSAE treatment had the best tensile property. The impact of GSAE on dough tensile properties may be related to the fact that GSAE is effective as a plasticizer on gluten protein, decreasing intermolecular interactions, increasing the number of hydrogen bonds, and enhancing the mechanical properties of the sample [42,43]. When the GSAE concentration was further increased, the breaking energy and extensibility of gluten protein reduced, demonstrating that excess anthocyanin caused a negative influence on gluten strength. It is speculated that during the complex formation process, phenolic substances and their oxidation products (quinones) may cross-link with amino and sulfhydryl side chains of protein polypeptide chains to form non-reducing covalent bonds such as C–N or C–S, forming polymer covalent polymers and eventually leading to the degradation of complex sample extensibility [44].

### 3.4. Creep and Recovery Properties

**Figure 3:**
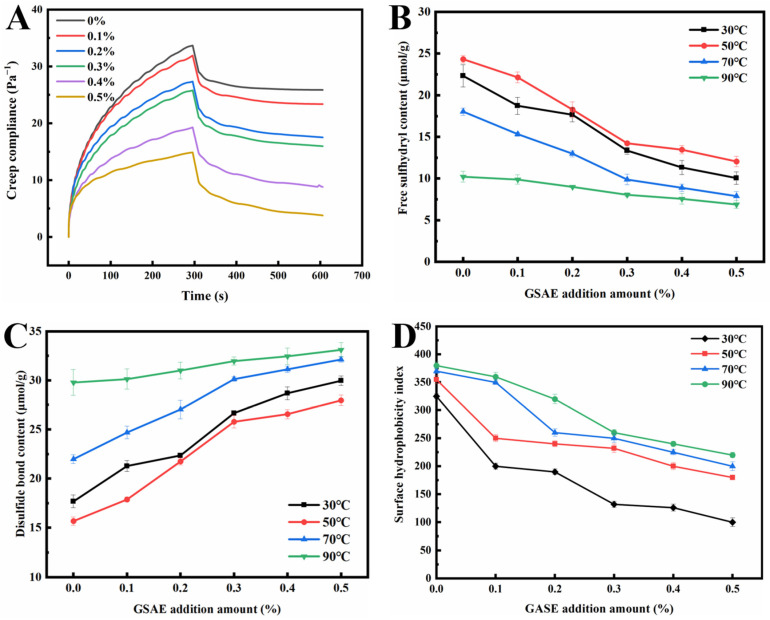
The creep and recovery curve (**A**), free sulfhydryl content (**B**), disulfide bond content (**C**), and surface hydrophobicity index (**D**) of gluten protein treated with different levels of GSAE.

The creep recovery test can directly reflect the rheological properties of gluten and dough, allowing for the evaluation of gluten viscoelasticity and dough quality [45]. The creep recovery curve of dough mainly consists of two stages: creep (0~about 300 s) and recovery (about 300 s~600 s) (Figure 3A). The creep recovery curve of dough presents nonlinear deformation in the creep range. The recovery period shows the characteristics of partial recovery, which characterizes the viscoelastic behavior of the dough during the extended time [46]. As shown in Figure 3A, the creep recovery curves of samples with and without GSAE introduction showed a similar trend, which was consistent with the creep recovery characteristics in previous reports [45,47]. The creep strain of dough decreased with increasing the GSAE addition in the creep stage (0~about 300 s), and dough with substantial deformation was more difficult to recover in the recovery stage. It might be due to the interaction between GSAE and a large number of hydrophobic sites in gluten protein, thereby enhancing gluten aggregation. Previous reports have proved that the augment of hydrophobic sites could increase the possibility of hydrophobic interactions between protein hydrophobic branch chains and lipid hydrophobic groups [48,49]. Nevertheless, Jiang, et al. [50] believe that the creep strain is closely related to the moisture content of the dough, and generally, the dough with higher moisture content has a larger maximum creep strain. Due to the higher number of hydroxyl groups, GSAE has higher water absorption, allowing more water molecules to bind to GSAE via hydrogen bonds, increasing competition for water between GSAE and other components (such as proteins and starches), and ultimately affecting the water distribution in the dough [51]. Additionally, when the amount of GSAE was gradually increased, the dough could recover to a stable state more quickly during the recovery stage, indicating that the introduction of GSAE enhanced the viscoelasticity and deformation resistance of the dough. It was speculated that GSAE induced the cross-linking of proteins in the dough, thus strengthening the network structure of the gluten protein [52].

### 3.5. Free SH and S-S Contents

The free SH content of dough is used to reflect the stability of the gluten network structure [53]. The free SH content in gluten protein with different temperature conditions and different GSAE addition amounts is shown in Figure 3B. The free SH content grew dramatically as the heating temperature climbed from 30 °C to 50 °C, which corresponded to the rheological test findings. It is possible that the increase in free SH content following 50 °C treatment resulted in a reduction in G′ [39]. On the other hand, the free SH content reduced as the amount of GSAE added increased at a heating temperature of 30 °C~50 °C. A possible explanation is that the reducibility of GSAE allows disulfide bonds in gluten to be reduced to free SH. The reducibility of GSAE is due to a large number of phenolic hydroxyl groups in its structure. These phenol groups participate in REDOX reactions by forming quinones and providing electrons, leading to an increase in the free SH content [1]. Han, et al. [54] also found that adding 1% tea polyphenols increased the free SH content, which was attributed to the reduction in disulfide bonds in gluten by tea polyphenols. However, the content of the free sulfhydryl group increased when the heating temperature was higher than 50 °C, indicating that the combination degree of GSAE and gluten protein increases due to the increase in temperature. Meanwhile, the hydrogen bond and hydrophobic interaction in the gluten protein network are enhanced, and the free sulfhydryl group is also gradually transformed into a disulfide bond.

The disulfide bond is one of the most important components in the structural and functional properties of gluten proteins, which is crucial for gluten proteins to form a viscoelastic network structure in wheat flour dough [55]. The change of disulfide bond content in dough will affect the formation of gluten network structure and ultimately influence the quality of dough products [56]. It can be observed in Figure 3C that with the increase of GSAE, the content of the disulfide bond in gluten increased in a dose-dependent manner, which is opposite to the trend of changes in free sulfhydryl content. Therefore, due to the addition of GSAE, there are more intra-chain hydrogen bonds in the gluten protein network, which promotes the extension and arrangement of protein–peptide chains, thereby increasing the content of disulfide bonds and promoting the formation of intermolecular hydrogen bonds [39].

### 3.6. Surface Hydrophobicity

In order to investigate whether GSAE can use aromatic rings to attach to protein surfaces or cross-link with different protein molecules [1], the surface hydrophobicity of dough was measured, and the results are shown in Figure 3D. The surface hydrophobic index of gluten protein rose with increasing heat treatment temperature under the same GSAE concentration. It could be because heating destroys the hydrophobic connection between proteins, exposing more hydrophobic groups to the internally folded protein molecules, which subsequently attach to fluorescent probes, increasing the surface hydrophobicity index [57]. The surface hydrophobicity index reduced in samples treated with GSAE compared to CK at the same temperature, showing that the addition of GSAE caused the aggregation of gluten proteins. As shown in Figure 3D, under heating temperatures of 30 °C, 50 °C, 70 °C, and 90 °C, the surface hydrophobicity of gluten protein reduced by 42.1%, 45.9%, 21.1%, and 69.2%, respectively, as the GSAE concentration increased from 0% to 0.5%, which might be due to the intermolecular cross-linking between GSAE and gluten protein and led to the change of its conformation. There might be two causes: on the one hand, GSAE directly binds to the hydrophobic sites of gluten proteins, and on the other hand, the intermolecular interaction between gluten proteins and GSAE indirectly leads to conformational changes in protein molecules [1].

### 3.7. Microstructure

**Figure 4:**
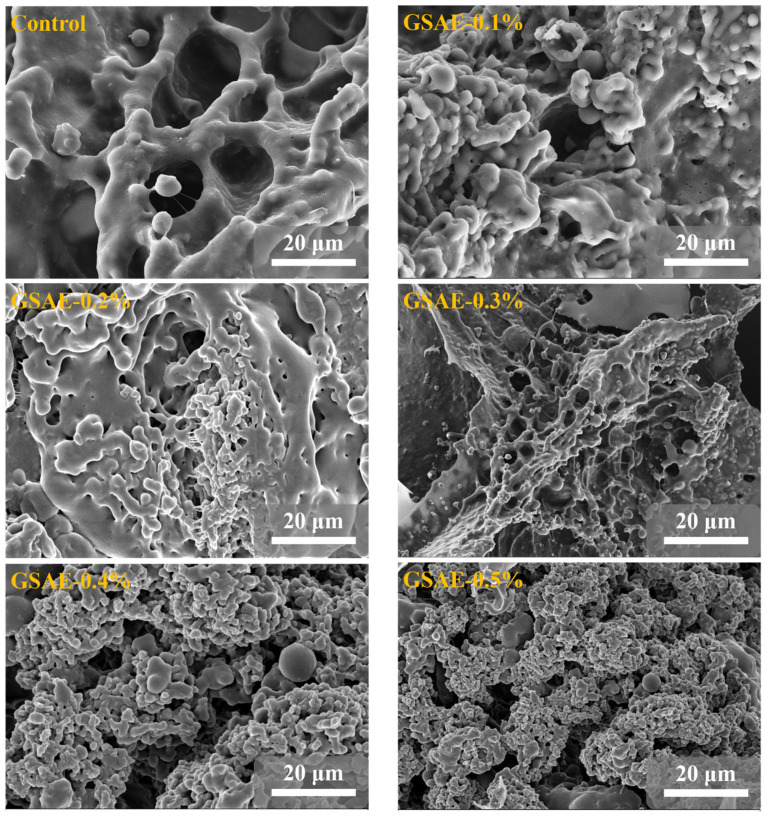
SEM images of gluten microstructure treated with different levels of GSAE.

In order to understand the effect of GSAE on dough microstructure, SEM was used to observe dough structure. Gluten, as depicted in Figure 4, is primarily composed of glutenin and melolin, which form a network structure that is entangled with one another. Among them, glutenin is relatively large, and the skeleton of gluten protein runs through the whole network structure of gluten protein, showing a continuous fiber shape, which plays the role of supporting dough; while melolin is spherical and interspersed in the fibers [39]. As shown in Figure 4, the gluten protein without GSAE treatment (CK) has a smooth surface, large voids in the network structure, and a long distance between the fibers. Compared with CK, the gaps in the gluten skeleton after GSAE treatment decreased in a dose-dependent manner with the increase of GSAE concentration, the number of gluten-like regions increased significantly, and the binding between proteins became closer. These findings suggest that adding GSAE to the gluten network enhances it by making the gluten microstructure more continuous and denser. Chen, Ni, Thakur, Wang, Zhang, Shang and Wei [23] also discovered that the non-covalent interaction between polyphenol in grape seed meal and gluten protein resulted in the formation of a denser network structure of gluten protein. According to the findings, the introduction of GSAE stimulated gluten protein aggregation, promoted the intermolecular aggregation or crosslinking of gluten protein, and improved the structure of gluten networks [1].

## 4. Conclusions

The effects of GSAE on the microstructure and physicochemical properties of wheat gluten protein were investigated. The results demonstrated that the network structure of gluten protein was damaged by heat treatment, while the introduction of GSAE helped to maintain gluten network and enhance viscoelasticity. The heat treatment also destroyed the hydrophobic interaction between GSAE and gluten, while GSAE increased the thermal denatalization temperature and enhanced the thermal stability of gluten. Furthermore, the GSAE treatment promoted the conversion of free sulfhydryl groups to disulfide bonds in gluten proteins, strengthened the hydrogen bond links in the chains, and promoted the chain extension and arrangement, which contribute to the crosslinking and stability of the gluten network. The gluten protein cross-linked with GSAE had more dense structure and clearer layer structure, which was beneficial to the stability of the gluten network. Although GSAE shows potential applications, further studies are needed to fully elucidate the effects of anthocyanins on gluten processing and structural properties under more complex food processing systems. Our findings imply that GSAE has some promise as a quality enhancer added to wheat products, which might be a promising research area for reducing the prevalence of chronic illnesses.

## Figures and Tables

**Table 1:** Denaturation temperature (Tp) and denaturation enthalpy (ΔH) of gluten protein treated with different levels of GSAE.

GSAE (%)	30 °C		50 °C		70 °C		90 °C	
	Tp	ΔH	Tp	ΔH	Tp	ΔH	Tp	ΔH
	(°C)	(J/g)	(°C)	(J/g)	(°C)	(J/g)	(°C)	(J/g)
0	75.52	216.6	73.55	220.8	74.50	178.4	75.62	160.5
0.1	73.45	174.4	75.32	193.4	75.12	169.1	76.35	152.4
0.2	75.43	169.3	77.65	173.6	78.29	160.2	78.68	147.6
0.3	77.37	152.6	78.49	163.2	78.96	150.3	79.36	135.8
0.4	78.90	148.2	79.28	142.3	79.56	145.9	80.69	128.6
0.5	79.54	143.6	80.65	135.6	80.98	128.9	82.69	114.3

**Table 2:** The tensile properties of gluten protein treated with different levels of GSAE.

GSAE Addition (%)	Stretching Energy (g)	Breaking Distance Starting from 30 min (mm)	Fracture Energy (g.s)	Extensibility (mm)
0	8.1 ± 0.1 ^e^	31.6 ± 3.2 ^d^	190.3 ± 2.0 ^a^	29.2 ± 2.0 ^c^
0.1	10.3 ± 3.0 ^de^	37.3 ± 7.0 ^cd^	251.0 ± 2.0 ^b^	34.0 ± 3.0 ^c^
0.2	14.4 ± 2.0 ^c^	39.0 ± 3.6 ^c^	327.7 ± 3.0 ^c^	34.2 ± 1.9 ^c^
0.3	24.1 ± 1.0 ^a^	65.3 ± 1.9 ^a^	808.0 ± 5.0 ^d^	61.6 ± 5.1 ^a^
0.4	17.7 ± 0.7 ^b^	52.4 ± 1.1 ^b^	400.2 ± 2.1 ^e^	45.2 ± 2.0 ^b^
0.5	13.3 ± 2.0 ^cd^	36.0 ± 1.5 ^c^	387.1 ± 2.8 ^f^	40.2 ± 2.0 ^b^

Note: data expressed as mean ± SD of triplicate measurements, values within the column followed by different letters indicate a significant difference (*p* < 0.05).

## Data Availability

Data is contained within the article.
